# Clinical characteristics of cardiovascular patients with extremely low levels of high-density lipoprotein cholesterol

**DOI:** 10.1186/s12944-021-01583-w

**Published:** 2021-10-30

**Authors:** Lufan Sun, Lian Duan, Dalin Jia

**Affiliations:** Department of Cardiology, The First Hospital of China Medical University, 155 North Nanjing Street, 110001 Shenyang, Liaoning Province China

**Keywords:** High-density lipoprotein cholesterol, Extreme, Low, Inflammation, Adverse factor

## Abstract

**Background:**

Extremely low levels of high-density lipoprotein cholesterol (HDL-C) are related to high cardiovascular mortality. The underlying mechanism is not well known. This research aims to study the clinical characteristics of cardiovascular patients with extremely low levels of HDL-C.

**Methods:**

All cardiovascular patients in a single Chinese cardiology center that were admitted from January to December 2019 were reviewed. The clinical characteristics of those with HDL-C<20 mg/dL were investigated.

**Results:**

A total of 20,655 individuals were enrolled. Of these, 52.17 % were males, and the average age was 58.20 ± 12.98 years old. The prevalence of HDL-C<20 mg/dL was 0.47 % for all patients (*N*=98) and 1.05 % for inpatients. Of those with HDL-C<20 mg/dL, 88.8 % were inpatients, and 77.6 % were males. Their average age was 60.7 ± 15.1 years. Compared with matched patients with normal HDL-C, systemic inflammation (OR= 5.556, 95% CI 2.798–11.030), hypoalbuminemia (OR=5.714, 95% CI 2.702–12.085), hyperuricemia (OR=5.156, 95% CI 2.560–10.386), low T3 syndrome (OR=4.278, 95% CI 1.627–11.245), anemia (OR=3.577, 95% CI 1.680–7.617), diabetes (OR=3.534, 95% CI 1.693–7.376) and hypertriglyceridemia (OR=2.493, 95% CI 1.264–4.918) were identified as adverse concomitant factors of extremely low HDL-C. HDL-C levels were inversely correlated with the total risk scores in patients with HDL-C<20 mg/dL (*r*=-0.381, *P*<0.001) and more significantly correlated in patients with HDL-C<15 mg/dL (*r*=-0.511, *P*=0.004).

**Conclusions:**

Extremely low levels of HDL-C tend to occur more frequently in males, older individuals and inpatients. For cardiovascular patients, extremely low levels of HDL-C are usually due to the presence of multiple adverse factors with relatively severe conditions. This could explain the high cardiovascular mortality of individuals with extremely low levels of HDL-C.

## Background

The relationship between high-density lipoprotein cholesterol (HDL-C) levels and cardiovascular deaths has been demonstrated to be a U-shaped curve [1]. Such a relationship indicates that both extremely low and extremely high levels of HDL-C are potentially harmful. Low HDL-C was associated with higher mortalities compared with high HDL-C [2]. Thus, the cardiovascular mortality of those with extremely low HDL-C is highest. It is necessary to investigate the underlying mechanisms associated with extremely low levels of HDL-C. Although the prevalence of low HDL-C levels has been reported to be very high in different countries and regions [3–5], extremely low HDL-C levels, which are defined as HDL-C<20 mg/dL (0.52 mmol/L), were definitely rare [6].

Mutations of ATP-binding cassette transporter A1, lecithin: cholesterol acyltransferase or apolipoprotein A-I, are known genetic causes of extremely low or even undetectable levels of HDL-C [7]. They are rare inherited disorders leading to defects in HDL formation or maturation. In the general population, acquired factors account for approximately half of the incidences of extremely low HDL-C [6]. Severe hypertriglyceridemia, especially when serum triglycerides are over 500 mg/dL, may lead to very low HDL-C [8]. Marked reduction of HDL-C was described previously in patients with cholestatic liver disease, which suppressed elements of HDL assembly and maturation [6]. Specific medications, such as androgenic anabolic steroids and peroxisome proliferation activated receptor (PPAR) agonists, including thiazolidinedione and fibrates, were also found to cause extremely low levels of HDL-C [9]. Some lymphoproliferative diseases have been shown to sharply decrease HDL-C levels because of the simultaneous elevation in paraprotein or cytokine levels [6, 10]. The factors listed above were related to severe HDL-C deficiency. In addition, diabetes, obesity and smoking are some common factors leading to mild and moderate decreases in HDL-C [3, 4, 11, 12].

Notably, individuals with genetic defects that lead to extremely low HDL-C levels are not always susceptible to cardiovascular diseases [13]. Large-scale Mendelian randomization studies proved that gene mutations attributed to changes in HDL-C concentration were not related to coronary atherosclerotic disease [14, 15]. It is suspected that the high mortality of extremely low HDL-C should be determined by phenotypes or other relevant concomitant factors. To date, little is known about the clinical characteristics that are exclusive to cardiovascular patients with extremely low HDL-C. Therefore, this research aims to study the relevant concomitant factors of cardiovascular patients with extremely low HDL-C levels in a single cardiology center.

## Methods

### Study population

Cardiovascular patients were retrospectively recruited from the Department of Cardiology at the First Hospital of China Medical University. To avoid differential effects caused by the season in which the cardiovascular patients were admitted, patients from January to December 2019 were chosen. Both outpatients and inpatients were included. Repeated HDL-C tests of the same subjects were excluded, except the first test. Patients under the age of 18 years were also excluded. Personal information, including sex, age, diagnosis and HDL-C level, was recorded for each subject.

Because detailed information could not be acquired from outpatients, only inpatients were further evaluated. For inpatients with HDL-C<20 mg/dL, the levels for total cholesterol (TC), low-density lipoprotein cholesterol (LDL-C), triglyceride (TG), serum albumin, uric acid, estimated glomerular filtration rate (eGFR), hemoglobin, high-sensitivity C-reactive protein (hsCRP), free thyroxine (FT4), free triiodothyronine (FT3), thyroid stimulating hormone (TSH), glycosylated hemoglobin (HbA1C), as well as body mass index (BMI) on admission were also reviewed from hospitalization records. Medical history, specific diseases (cholestatic liver disease, acute hepatitis and HIV infection) and ongoing medications (androgenic steroids, thiazolidinediones and fibrates) were checked. They were paired with matched inpatients with normal levels of HDL-C (40–60 mg/dL) by sex, age and chief diagnosis from the same database. These matched patients with normal levels of HDL-C were set as the control group. The study was approved by the ethics committee of the hospital in accordance with the Declaration of Helsinki. Because this was a retrospective observational study without any interventional procedure on patients, written informed consent of the investigated subjects was exempted (Approved No. 2020307).

### HDL-C measures

All blood samples were centrally tested in the clinical laboratory of the hospital. HDL-C levels were determined by a direct assay (Kyowa Medex, Japan) on a COBAS 8000 automatic biochemical analyzer (Roche, Germany). Because lipid profiles were reported in mmol/L, they were converted to mg/dL to adhere to international guidelines by calculation (for triglycerides, 1 mmol/L = 88.57 mg/dL, and for cholesterol, 1 mmol/L = 38.67 mg/dL).

### Evaluations of relevant concomitant factors

Several relevant conditions were selected as concomitant factors of extremely low levels of HDL-C, including hypercholesterolemia, hypertriglyceridemia, systemic inflammation, hypoalbuminemia, hyperuricemia, renal dysfunction, anemia, low T3 syndrome, obesity, diabetes, current smoking status, alcohol consumption and critical conditions. The definitions of these factors are presented in Table 1. Factors that were demonstrated to increase the possibility of extremely low levels of HDL-C were considered final adverse factors. Each adverse factor was given a score of 1. Because the levels of TG and hsCRP both followed a skewed distribution, an additional score would be given respectively when hsCRP ≥ 10.0 mg/L and TG ≥ 200 mg/dL. The total risk score was calculated as the sum result of all the adverse factors for each individual.

**Table 1:** Definitions of relevant concomitant factors with extremely low HDL-C

Concomitant factors	Definition
**Hypercholesterolemia**	TC ≥200 mg/dL and/or LDL-C ≥130 mg/dL
**Hypertriglyceridemia**	TG ≥150 mg/dL
**Systemic inflammation**	hsCRP > 3.0 mg/L
**Hypoalbuminemia**	Serum albumin < 35 g/L
**Hyperuricemia**	Uric acid ≥ 417umol/L for male or uric acid ≥ 357umol/L for female
**Renal dysfunction**	eGFR < 60 ml/min/1.73m^2^
**Anemia**	Hemoglobin < 120 g/L for male or hemoglobin < 110 g/L for female
**Low T3 syndrome**	FT3 < 2. 63pmol/L with normal FT4 and TSH
**Obese**	BMI ≥ 28 kg/m^2^
**Diabetes**	Prior history or HbA1C ≥ 6.5 %
**Current smoking**	Smoking within 3 recent months
**Alcohol consumption**	Daily drinking of alcohol
**Critical condition**	MODS

*HDL-C* high-density lipoprotein cholesterol; *TC* total cholesterol; *LDL-C* low-density lipoprotein cholesterol; *TG* triglyceride; *hsCRP* high-sensitive C-reactive protein; *eGFR* estimated glomerular filtration rate; *T3* triiodothyronine; *FT3* free triiodothyronine; *FT4* free thyroxine; *TSH* thyroid stimulating hormone; *BMI* body mass index; *HbA1C* glycosylated hemoglobin; *MODS* multiple organ dysfunction syndrome

### Statistical analysis

Data analysis was performed with SPSS 23.0 software. Categorical data were expressed as case numbers and percentages. Continuous data were expressed as median (minimum, maximum) for skewed distribution or the mean ± standard deviation for normal distribution. A trend test was performed to examine the linear trend between HDL-C level and age. Differences between groups of the extremely low HDL-C and the controls were compared by Student’s t-test or nonparametric u-test for continuous variables. Univariate correlations between HDL-C levels and different parameters were evaluated by Pearson analysis or Spearman analysis. Multivariate linear regressions were performed to determine independent factors. To examine odds ratios (ORs) of the concomitant factors for those with extremely low HDL-C levels, logistic regression analysis was conducted. *P*<0.05 was set as a significant standard in statistics.

## Results

### General clinical characteristics of participants

The final analysis was composed of 8255 inpatients and 12,400 outpatients, with a total of 20,655 individuals. There were 10,775 males (52.17 %) and 9880 females (47.83 %). Their average age was 58.20 ± 12.98 years. The HDL-C level ranged from 3.9 mg/dL to 179.4 mg/dL. The mean HDL-C level was 46.0 ± 13.5 mg/dL. The HDL-C level of inpatients was much lower than that of outpatients (40.6±11.6 mg/dL vs. 49.5±13.5 mg/dL, *P*<0.001). Besides, the HDL-C levels of males were lower than levels of females (41.8±12.0 mg/dL vs. 50.7±13.9 mg/dL, *P*<0.001). Levels of HDL-C presented an ascending trend with aging in male outpatients, female outpatients and male inpatients. Levels of HDL-C presented a descending trend with aging in female inpatients (Table 2).

**Table 2:** Linear trends of HDL-C levels with aging(mean ± standard deviation, mg/dL)

Age (years)	<40	40-49	50-59	60-69	70-79	≥80	*P* for trend
**Outpatients**							
Male	42.2±10.4	43.3±11.2	45.2±12.0	46.4±12.4	48.0±13.1	49.1±13.5	<0.001
Number of cases	995	1064	1650	1632	521	151	
Female	53.0±13.5	53.0±13.5	53.4±13.5	53.4±13.1	54.9±14.7	55.7±15.1	<0.001
Number of cases	500	902	1971	2055	761	198	
**Inpatients**							
Male	35.6±10.1	35.6±8.9	37.1±10.1	38.3±10.1	39.1±11.2	39.1±12.4	<0.001
Number of cases	335	550	1254	1715	678	230	
Female	46.8±13.1	46.8±13.9	45.6±11.6	44.9±11.6	44.1±12.0	44.5±12.8	0.004
Number of cases	142	201	775	1340	777	258	

*HDL-C* high-density lipoprotein cholesterol

### Characteristics of patients with extremely low levels of HDL-C

The percentages of patients with HDL-C levels under 40 mg/dL, 20 mg/dL and 15 mg/dL were 36.83 % (*n*=7607), 0.47 % (*n*=98) and 0.17 % (*n*=36), respectively. The average age of patients with HDL-C<20 mg/dL was 60.7 ± 15.1 years. Among the 98 patients with HDL-C<20 mg/dL, there were 87 inpatients (88.8 %) and 76 were male (77.6 %).

The percentage of inpatients with HDL-C levels under 20 mg/dL was 1.05 %. For inpatients, the main causes of hospitalization were acute coronary syndrome (*n*=50, 57.5 %), congestive heart failure (*n*=21, 24.1 %) and acute myocarditis (*n*=7, 8.0 %). There was no detected lymphoproliferative disease or use of androgenic anabolic steroids. A patient with cholestatic liver disease, a patient with fenofibrate medication and a third patient with triglycerides over 500 mg/dL were excluded from comparison and scoring, in case these conditions made analysis confusing.

**Fig. 1:**
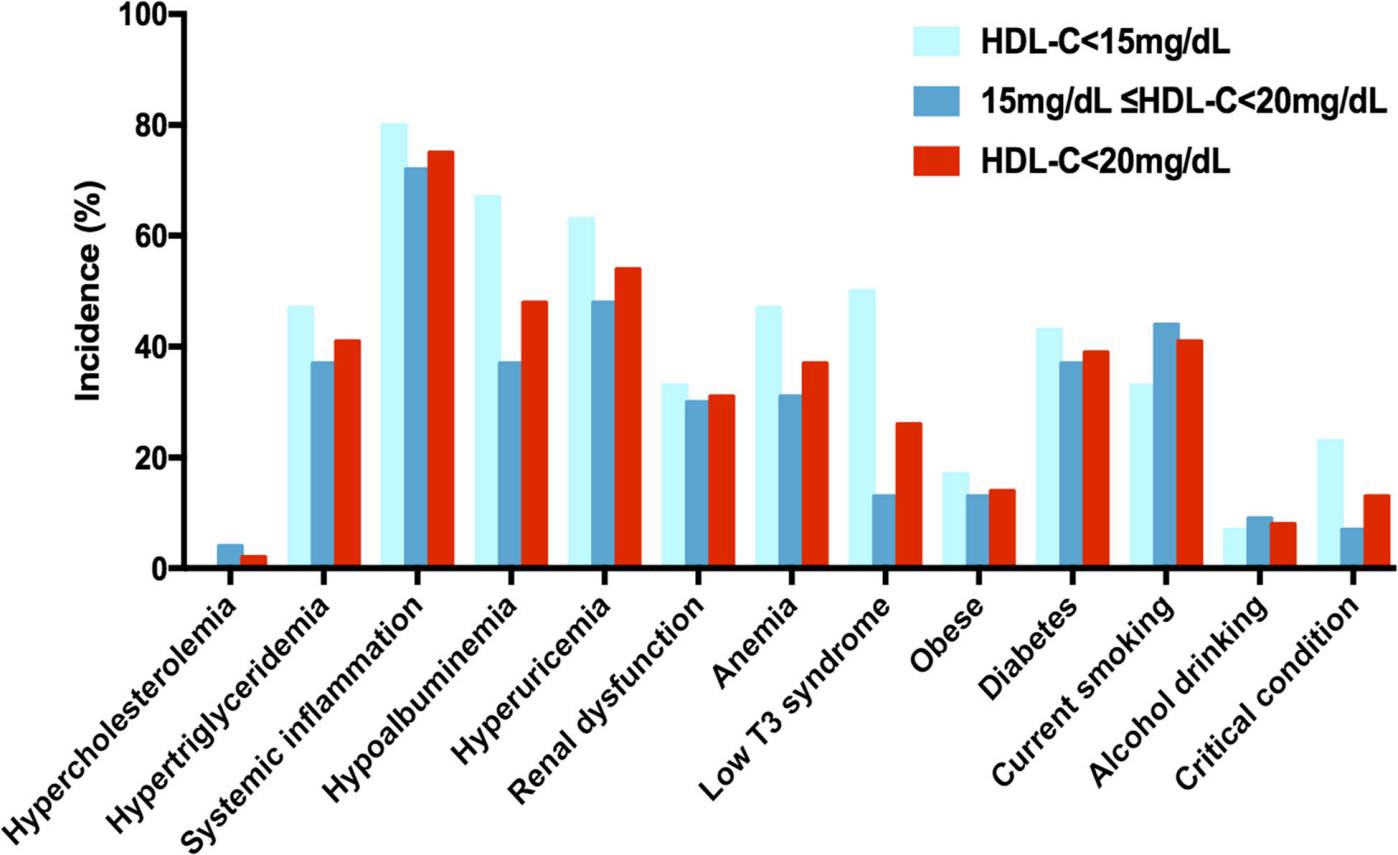
Incidences of relevant concomitant factors in inpatients with extremely low HDL-C. For HDL-C<15 mg/dL, the case number was 30. For 15 mg/dL ≤HDL-C<20 mg/dL, the case number was 54. For HDL-C<20 mg/dL, the case number was 84

In the remaining 84 inpatients, the percentage of each relevant concomitant factor is presented in Fig. 1. The top three factors observed in inpatients with HDL-C<20 mg/dL were systemic inflammation (75.0 %), hyperuricemia (53.6 %) and hypoalbuminemia (47.6 %). For inpatients with HDL-C<15 mg/dL, the top three factors were systemic inflammation (80.0 %), hypoalbuminemia (66.7 %) and hyperuricemia (63.3 %). However, the top three factors for inpatients with HDL-C levels of 15-20 mg/dL were systemic inflammation (72.2 %), hyperuricemia (48.1 %) and smoking (44.4 %). In the univariate analysis, HDL-C levels were negatively correlated with hsCRP levels and positively correlated with LDL-C, serum albumin and FT3 levels. Parameters, such as age, TG, uric acid, eGFR, hemoglobin, BMI and HbA1C, did not correlate with HDL-C levels. The levels of LDL-C and FT3 were independent factors associated with HDL-C levels according to multivariate regression (Table 3).

**Table 3:** Linear correlations and regressions between clinical parameters and HDL-C level in inpatients with extremely low HDL-C (*N*=84)

	Univariate Correlation		Multivariate Regression	
	**r**	***P***	**β**	***P***
**Age(years)**	-0.022	0.843	0.292	0.093
**LDL-C(mg/dL)**	0.383	<0.001	0.818	0.009
**TC(mg/dL)**	0.185	0.092	-0.617	0.036
**TG(mg/dL)**	-0.090	0.416	-0.030	0.854
**hsCRP(mg/L)**	-0.296	0.007	0.097	0.518
**Serum albumin(g/L)**	0.369	<0.001	0.152	0.332
**Uric acid(umol/L)**	-0.015	0.897	-0.048	0.723
**eGFR(ml/min/1.73m**^**2**^**)**	-0.061	0.620	-0.070	0.741
**Hemoglobin(g/L)**	0.205	0.063	0.053	0.640
**FT4(pmol/L)**	0.182	0.097	0.042	0.717
**FT3(pmol/L)**	0.460	<0.001	0.462	0.011
**TSH(mIU/L)**	-0.090	0.416	-0.114	0.278
**BMI(kg/ m**^**2**^**)**	-0.169	0.179	-0.122	0.377
**HbA1C(%)**	-0.088	0.436	0.039	0.715

*HDL-C* high-density lipoprotein cholesterol; *TC* total cholesterol; *LDL-C* low-density lipoprotein cholesterol; *TG* triglyceride; *hsCRP* high-sensitive C-reactive protein; *eGFR* estimated glomerular filtration rate; *FT4* free thyroxine; *FT3* free triiodothyronine; *TSH* thyroid stimulating hormone; *BMI* body mass index; *HbA1C* glycosylated hemoglobin. r represented coefficient of univariate correlation between HDL-C levels and parameters. *β* represented standard coefficient of multivariate regression between HDL-C levels and parameters

### Differences between inpatients with extremely low HDL-C levels and their counterparts with normal HDL-C levels

Inpatients with extremely low levels of HDL-C also exhibited lower levels of cholesterol, serum albumin, hemoglobin and FT3, compared with their counterparts with normal HDL-C. Conversely, they exhibited higher TG, hsCRP, uric acid, FT4, and HbA1C levels and a higher BMI. There was no difference in eGFR or TSH levels (Table 4). Systemic inflammation, hyperuricemia and hypoalbuminemia significantly increased the possibility of extremely low HDL-C levels to over five-fold of that of normal HDL-C levels. In addition, low T3 syndrome, anemia, diabetes and hypertriglyceridemia were also revealed to increase the likelihood of extremely low HDL-C. Hypercholesterolemia and alcohol consumption reduced the possibility of extremely low HDL-C (Table 5).

**Table 4:** Comparisons between inpatients with extremely low HDL-C and normal HDL-C

	Low Group (*N*=84)	Normal Group (*N*=84)	*P*
**HDL-C(mg/dL)**	15.9±4.1	48.8±5.4	<0.001
**LDL-C(mg/dL)**	72.6±28.6	104.5±35.6	<0.001
**TC(mg/dL)**	118.6±30.7	170.4±40.3	<0.001
**TG(mg/dL)**	125.3 (46.1, 435.8)	93.9 (19.5, 265.7)	<0.001
**hsCRP(mg/L)**	15.60 (0.15, 402)	1.89 (0.15, 190)	<0.001
**Serum albumin(g/L)**	35.2±6.4	39.3±5.2	<0.001
**Uric acid(umol/L)**	454±186	328±99	<0.001
**eGFR(ml/min/1.73m**^**2**^**)**	84.35±39.48	88.45±38.26	0.521
**Hemoglobin(g/L)**	125±24	136±21	0.003
**FT4(pmol/L)**	13.80±2.52	12.98±1.73	0.015
**FT3(pmol/L)**	3.17±0.83	3.93±0.75	<0.001
**TSH(mIU/L)**	1.63±1.39	1.92±1.28	0.163
**BMI(kg/ m**^**2**^**)**	25.22±3.97	23.78±3.47	0.024
**HbA1C(%)**	6.8±1.4	6.0±0.9	<0.001

*HDL-C* high-density lipoprotein cholesterol; *TC* total cholesterol; *LDL-C* low-density lipoprotein cholesterol; *TG* triglyceride; *hsCRP* high-sensitive C-reactive protein; *eGFR* estimated glomerular filtration rate; *FT4* free thyroxine; *FT3* free triiodothyronine; *TSH* thyroid stimulating hormone; *BMI* body mass index; *HbA1C* glycosylated hemoglobin. Data were expressed as mean ± standard deviation for normal distribution or median (minimum, maximum) for skewed distribution

**Table 5:** Logistic regressions of relevant concomitant factors for inpatients with extremely low HDL-C

	OR (95% CI)	*P*
**Hypercholesterolemia**	0.065 (0.015 - 0.285)	<0.001
**Hypertriglyceridemia**	2.493 (1.264 – 4.918)	0.008
**Systemic inflammation**	5.556 (2.798 – 11.030)	<0.001
**Hypoalbuminemia**	5.714 (2.702 – 12.085)	<0.001
**Hyperuricemia**	5.156 (2.560 – 10.386)	<0.001
**Renal dysfunction**	1.589 (0.763 – 3.308)	0.216
**Anemia**	3.577 (1.680 – 7.617)	<0.001
**Low T3 syndrome**	4.278 (1.627 – 11.245)	0.003
**Obese**	1.833 (0.684 – 4.914)	0.228
**Diabetes**	3.534 (1.693 – 7.376)	0.001
**Current smoking**	1.916 (0.997 – 3.682)	0.051
**Alcohol consumption**	0.386 (0.150 – 0.995)	0.049
**Critical condition**	2.381 (0.789 - 7.181)	0.124

*HDL-C* high-density lipoprotein cholesterol; *T3* triiodothyronine ; *OR* odds ratio; *CI* confidence interval

ORs and CIs were calculated for extremely low HDL-C group by making normal group as a reference. Each group included 84 cases

### Correlations between risk scores and HDL-C levels

**Fig. 2:**
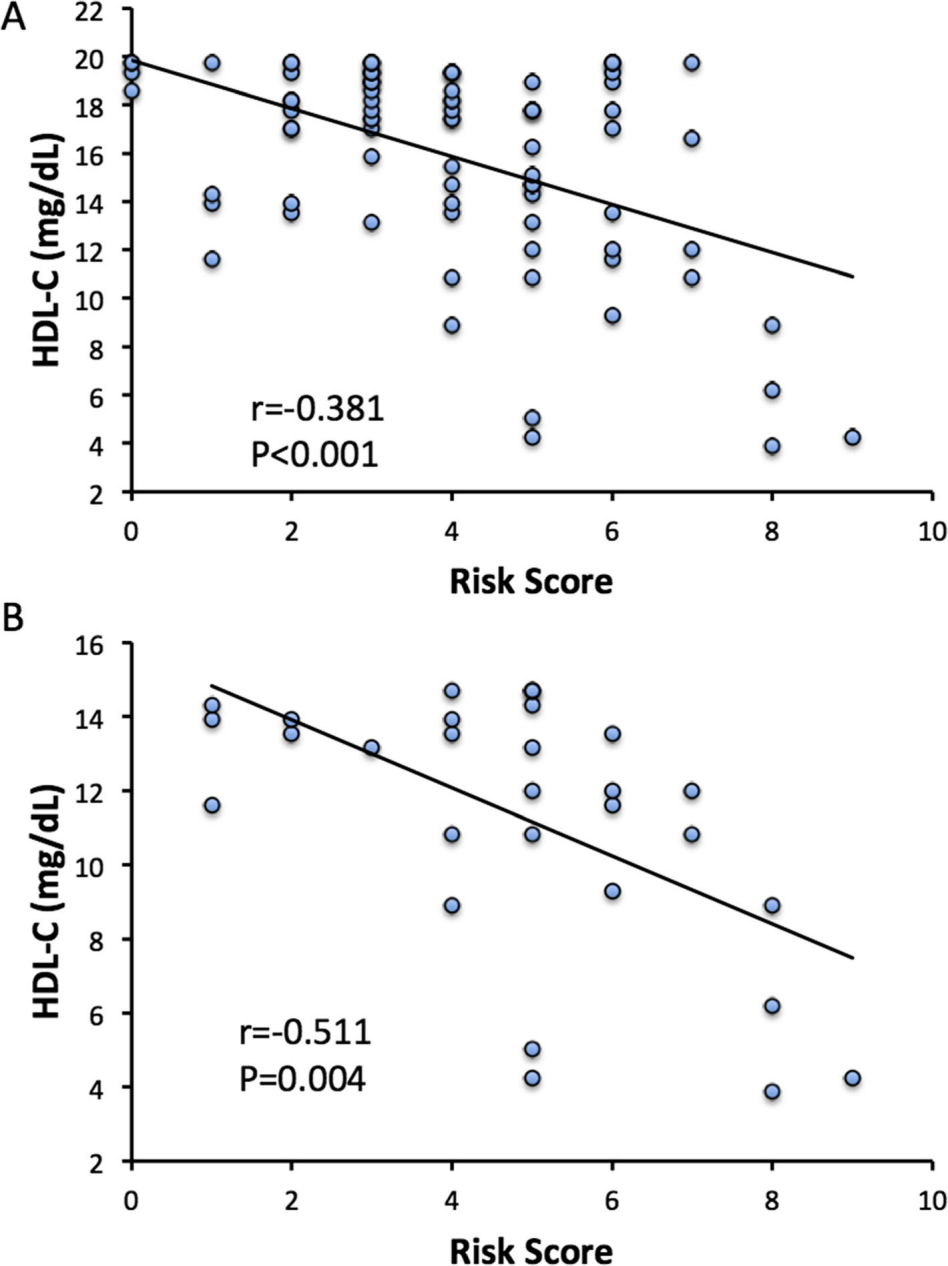
Reverse correlations between risk scores and HDL-C levels in inpatients with extremely low HDL-C. **A** Correlation between risk score and HDL-C level in inpatients with HDL-C<20 mg/dL (*N*=84). **B** Correlation between risk score and HDL-C level in inpatients with HDL-C<15 mg/dL (*N*=30)

The final adverse factors were systemic inflammation, hyperuricemia, hypoalbuminemia, low T3 syndrome, anemia, diabetes and hypertriglyceridemia, as shown in Table 5. All except four patients had at least one adverse factor. There were 66 patients (78.6 %) who scored ≥ 3 points, including 25 (83.3 %) in the HDL-C <15 mg/dL subgroup, and 41 (75.9 %) in the HDL-C 15–20 mg/dL subgroup. The HDL-C levels negatively correlated with the total risk scores in patients whose HDL-C levels were below 20 mg/dL (*r*= -0.381, *P*<0.001) (Fig. 2 A). In particular, the correlation became more significant in patients whose HDL-C levels were below 15 mg/dL (*r*=-0.511, *P*= 0.004) (Fig. 2B).

## Discussion

This observational study, for the first time, demonstrated several clinical characteristics of cardiovascular patients with extremely low levels of HDL-C. (1) These patients were likely to be males and inpatients. Although there was an ascending trend of HDL-C with aging in general, the relatively old age of these individuals implies that they might be more severely ill than the other individuals. (2) The common concomitant factors of extremely low levels of HDL-C were systemic inflammation, hyperuricemia, hypoalbuminemia and low T3 syndrome. These factors were essentially different from the common factors that were associated with mildly and moderately low HDL-C levels, which were hypercholesterolemia, smoking or obesity. (3) Extremely low HDL-C levels tended to be a comprehensive result of multiple adverse concomitant factors in cardiovascular patients. The HDL-C level might be reduced with severity and an increasing number of adverse concomitant factors.

### Comparisons with other studies and contributions of the current study

Sex-specific differences have been reported in both clinical manifestations and treatment outcomes [16]. The fact that extremely low HDL-C levels were more prevalent in males than females indicates the importance of exploring sex-specific approaches to the evaluation and treatment of HDL-C in the future. Low T3 syndrome is the state of decreased triiodothyronine with normal thyroxine and TSH, and it is an indicator of severe illnesses [17]. In this study, low T3 syndrome was found in half of the HDL-C<15 mg/dL cases, but it was not common in the HDL-C 15–20 mg/dL subgroup. This meant that the patients in HDL-C 15–20 mg/dL subgroup were in relatively better condition and might show factors that are similar to that of patients with moderate HDL-C levels, such as smoking, obesity and other common factors.

A high level of uric acid indicates an imbalance in purine metabolism with more production and less excretion. In patients with extremely low HDL-C levels, comparatively severe conditions might be a factor leading to fast catabolism and reduced renal function. Therefore, net accumulation of uric acid in circulation results in a high prevalence of hyperuricemia. Surprisingly, there was no significant reduction in renal function in this study. Therefore, hyperuricemia should be an independent factor associated with extremely low levels of HDL-C. A negative correlation of HDL-C and uric acid levels has been reported in previous studies [18, 19]. In contrast to metabolic syndrome, hyperuricemia was not parallel with obesity, hypertriglyceridemia or diabetes in those with extremely low HDL-C. Both hypoalbuminemia and anemia are signs of poor nutrition status. A cross-sectional study demonstrated that serum albumin ≤35 g/L independently predicted HDL-C<30 mg/dL in patients with acute coronary syndrome [20]. Although albumin might act as a potent cholesterol carrier and opponent of apolipoproteins [21, 22], a simultaneous lack of the main protein element of HDL, namely, apoA-I, might occur when albumin levels decrease. A similar explanation could involve low total cholesterol levels, which is supported by a positive association between albumin and total cholesterol levels in the US population [23].

It was reported that critical illnesses, such as severe sepsis, induced a decrease in HDL cholesterol content [24]. However, infection was not the only cause. Only a few inpatients in the current investigation had an acute infection. The systemic inflammation indicator hsCRP was notably elevated in the majority of patients to different extents. Forty-seven of the 84 patients in this study had severe systemic inflammation (hsCRP>10.0 mg/L). This implies a close relationship between inflammation and decreased HDL-C levels, probably in a dose-dependent manner. Even under lipid-lowering therapy, HDL-C and hsCRP levels maintained an inverse relationship [25]. There are two possible reasons. One reason is that the cholesterol efflux ability of HDL might be attenuated in an inflammatory cardiovascular background, resulting in a decrease in cholesterol content on HDL [26, 27]. The second reason is that, as the team of Alan reported, anti-inflammatory cytokines induced endocytosis of HDL and LDL into macrophages, leading to temporarily low HDL and LDL cholesterol levels in serum [28].

HDL-C level has been challenged time and time again for its association with cardiovascular diseases. Instead, the HDL function may be the real link with cardiovascular diseases [29]. Since the role of HDL in prevention and progression of cardiovascular diseases remains to be further clarified, we assume, in the current study, that extremely low level of HDL-C is only a marker, which reflects a comprehensive result of multiple adverse concomitant factors, and the high cardiovascular mortality is probably due to the presence of severe conditions. Previously, Mohsen and colleagues found that inflammation was an important factor associated with extremely low HDL-C levels [30]. HDL also exerts anti-inflammatory properties [31]. Furthermore, inflammation might be involved in hyperuricemia and hypoalbuminemia [32–34], and reduced HDL-C was repeatedly reported in inflammatory states, such as smoking, diabetes, obesity and even the recent COVID-19 [35, 36]. Thus, systemic inflammation, the most prevalent factor in this study, may be the core of all the adverse factors associated with the alterations of HDL cholesterol content. Our previous study demonstrated that the HDL-C/hsCRP ratio was superior to hsCRP in relation to left ventricular diastolic dysfunction [37], which was considered an inflammation-mediated abnormality [38]. Perhaps HDL-C should be considered a comprehensive inflammatory marker rather than a simple lipid value. Meanwhile, we could not deny the potential interrelationship among the concomitant factors being chosen in the study. Although as far as we can imagine, they are common clinical conditions that may be associated with HDL-C reduction, it is inevitable that these concomitant factors could be related with each other, as inflammation involves the link between cholesterol level and cardiovascular diseases [39]. Therefore, multivariate regressions between clinical parameters and HDL-C level were checked. The inflammatory parameter, which negatively correlated with HDL-C in univariate correlation, was not an independent parameter associated with HDL-C. This, in turn, implies that inflammation relates with other factors.

### Study strength and limitations

This study exclusively investigated cardiovascular patients with extremely low HDL-C levels for the first time and provides possible explanations for the high cardiovascular mortality of such unique individuals. However, several limitations should be noted.

Because this is a retrospective observational study conducted in a single center, the representativeness and persuasiveness of the results were limited. Thus, a causal relationship for each factor could not be established. In addition, the HDL-C levels of these patients at baseline and after treatment were not compared. In this case, the effect strengths of the adverse concomitant factors in this study were not definitely clear. Lipoprotein(a), the newly known lipid parameter associated with cardiovascular events [40], was not investigated. Thus, some useful information might be missing accordingly. Besides those specific medicines, which can induce sharp HDL-C reduction, lipid-modifying drugs, such as statins, may more or less impact the level of HDL-C and cardiovascular outcomes. Although we excluded cases with ongoing use of the specific medicines during the process of statistical analysis, we did not stratify cases according to other detailed lipid-modifying medications because of the small sample size. As a result, we could not conclude whether the results were attributed to cholesterol levels or effects of drugs, just like the concerns of Cholesterol Treatment Trialists’ Collaborators [41]. A similar investigation reported the clinical features of general patients with extremely low levels of HDL-C. It demonstrated that the proportion of cardiovascular diseases in these patients was much lower than that of malignancies [42]. This explains why the prevalence of extremely low HDL-C in the current study was lower than expected. Although over 20,000 patients were investigated, less than one hundred patients met the criteria. As a result, the size of samples was comparatively small. This study only serves as a pilot study, and the results still require further validation. According to the same study, nearly 99 % of patients had secondary causes, which is much higher than the proportion in the general population [42]. Although genetic causes were not identified, especially in the four individuals without any adverse concomitant factors, this study could still represent a majority of cases.

## Conclusions

The clinical characteristics of cardiovascular patients with extremely low levels of HDL-C are different from those commonly seen in individuals with mild and moderate HDL-C decrease. Extremely low levels of HDL-C tend to occur more frequently in males, relatively old individuals and inpatients. These low levels are usually due to the presence of multiple adverse factors with relatively severe conditions in cardiovascular patients. This could explain the high cardiovascular mortality rate of individuals with extremely low HDL-C levels. In the future, attention and special cares should be given to such patients to reduce mortality.

## Data Availability

All data generated and analysed during this study are included in this published article.

## Abbreviations

BMI: body mass index
eGFR: estimated glomerular filtration rate
FT3: free triiodothyronine
FT4: free thyroxine
HbA1C: glycosylated hemoglobin
HDL-C: high-density lipoprotein cholesterol
hsCRP: high-sensitive C-reactive protein
LDL-C: low-density lipoprotein cholesterol
PPAR: peroxisome proliferation activated receptor
TC: total cholesterol
TG: triglyceride
TSH: thyroid stimulating hormone
